# Surgical Management of Secondary Complex Microsurgical Reconstructions after Amputation and Severe Trauma Injuries: A Case Series

**DOI:** 10.3390/life14101303

**Published:** 2024-10-14

**Authors:** Marcel Hoh, Sebastian Geis, Silvan Klein, Lukas Prantl, Vadym Burchak, Juergen H. Dolderer

**Affiliations:** 1 Department of Plastic, Reconstructive, Aesthetic and Hand Surgery, University Medical Center, 95445 Bayreuth, Germanyjuergen.dolderer@klinikum-bayreuth.de (J.H.D.); 2 Department of Plastic and Reconstructive Surgery, University Regensburg, 93053 Regensburg, Germany

**Keywords:** reconstructive rocket, secondary procedures, complex hand surgery, functional outcomes, replantations, revascularisation

## Abstract

Introduction: Secondary complex microsurgical reconstructions after amputation and severe trauma injuries are often necessary to optimize functional outcomes. Methods and Patients: We reviewed eight patients who underwent extensive reconstruction after severe trauma. The details of secondary procedures are further described in the article. A literature search was performed using the National Center for Biotechnology Information (NCBI) database for studies evaluating secondary procedures after complex reconstructions. Discussion: To date, the order and the need for performing secondary procedures have yet to be fully defined. The tissues encountered include skin, soft tissue, bone, nerve, joint, and tendon. Conclusions: We described the use of a decision-theoretic approach to the secondary reconstruction. Treatment of a complex trauma should be measured by functional outcome.

## 1. Introduction

Plastic-surgical reconstruction of the extremities is based on the planning of the initial and secondary reconstruction. It needs both the principles of escalating complexity and interdisciplinary techniques [1].

Komatsu and Tamai described the first successful replantation of an amputated finger in 1965 [2,3]. 

Since then, the progress in microsurgical instrumentation and techniques increased the rate of successful replantation from less than 50% in early reports 46 to more than 90% [4,5].

However, replantation should not be considered successful until the function of the amputated part is restored; therefore, secondary surgical procedures are necessary to improve the final result [6].

These replanted parts often have many functional deficits and require more than one secondary procedure to recreate a near-normal function [6].

The causes of these functional deficits include, e.g., contractures, tendon adhesions, joint stiffness, malunion or nonunion of fractures, problems with nerval recovery, insufficient length recovery or imprecise opposition in case of an injured thumb and problems with soft tissue coverage [7,8,9,10,11,12,13,14].

However, the aesthetic outcome also plays a major role, especially for young female patients. 

The functional and psychological impacts of an amputation can highly affect the patient’s quality of life [15].

Therefore, the focus has shifted to functional outcome rather than viability, and secondary procedures play an increasing role in the patient management. The incidence of secondary operations varies from 2.6% to 91.7% [16,17,18,19,20,21,22,23,24].

Since nearly unlimited permutations and combinations of the severity, extent, and number of tissues are involved, the patterns of injuries are so variable that a standardized treatment is very difficult to establish [25].

Several techniques have been developed in order to achieve the goal of the recreation of a near-normal function [26,27]. Some techniques to treat destructed joints are arthrodesis, osteochondral grafting [28], costal cartilage grafts [29], perichondrial resurfacing [30,31], silicone arthroplasty [32,33], and endoprosthesis [33,34]. The incidence of tendon surgery is the highest.

Secondary operations after major limb replantations include flap procedures for coverage, corrective osteotomies and bone grafting, and the release of contractures and transfer of nerve, tendon, or muscle [35].

A large number of defects require free microvascular tissue transfer or even a complex reconstruction with chimeric flaps [6].

Infection [10,36], paresthesia [15], hypersensitivity [36], and problems with the blood supply are only a few reasons for secondary amputation [36,37,38,39]; however, reports of secondary amputation are rare.

There are multiple assessment tools and algorithms for the initial treatment management [40,41,42] but few reports have focused on the order and appropriateness in which secondary procedures should be performed [43,44].

Wang et al. designed a decision-theoretic approach for determining the order of performing secondary procedures [2]. However, such a decision procedure has not been affirmed by further reports and still depends on the surgeon’s experience after a detailed evaluation and discussion of the procedures and their outcomes with the patient. 

With a high level of surgical experience and forward planning, even the most difficult requirements of the reconstruction can be successfully mastered [1].

Based on eight major cases, we evaluated the current algorithms in order to establish a standardized treatment within secondary procedures. 

## 2. Material and Patients

An overview of the following cases can be seen in Table 1. 

### 2.1. Patient 1

**Figure 1:**
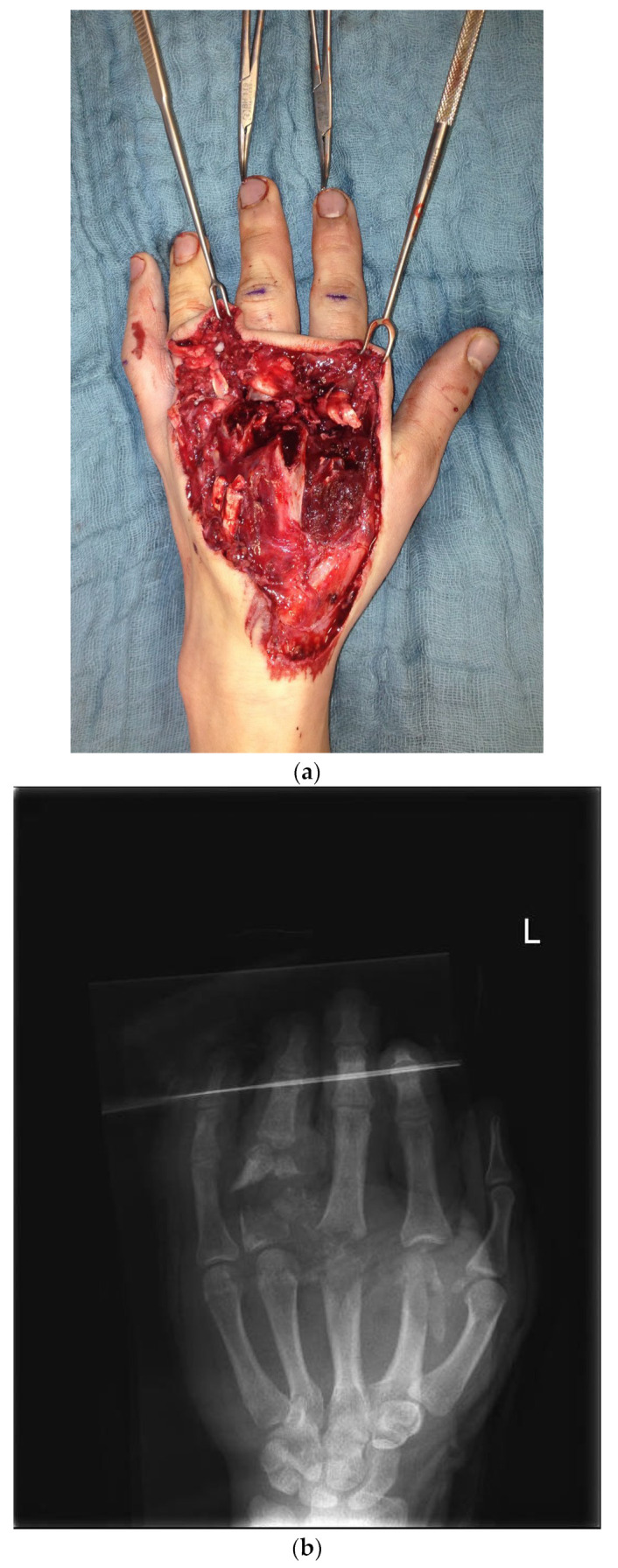

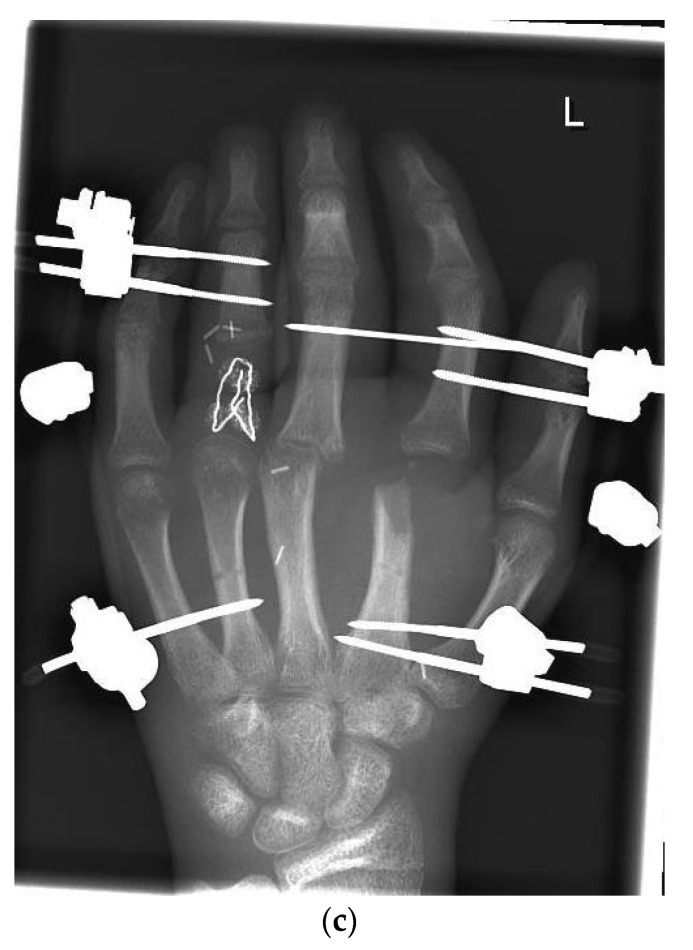
(**a**) Preoperative wound with exposed bone defect at MCP II–IV. (**b**). Preoperative X-ray with the destruction of MCP II–IV. (**c**) X-ray after the first surgical treatment using external fixators.

The first case shows a left-handed 17-year-old male patient with a buzz saw injury with a subtotal amputation of DII–DIV at the level of the MCP joints (Figure 1a). The preoperative X-ray showed a comminuted fracture of the proximal phalanx of DIV and destructed MCP joints of DII, DIII, and DIV (Figure 1b). 

During the initial operation, DIV was revascularized through the reconstruction of both nerves and arteries using vein grafts. A suture of the FDP tendon was performed as well as soft tissue restoration using skin from the forearm. In addition, a DIII-IV extensor suture was made, an external fixator was attached, and a mini septopal chain was inserted (Figure 1c).

Because of the comminuted fractures of the basal phalanx DIV and the distal metacarpals DII and DIII, an initial reconstruction was not possible, and we decided to perform complex reconstructions over time at the patient’s request. 

The patient was discharged after 3 weeks of follow-up treatment and ergotherapy.

**Figure 2:**
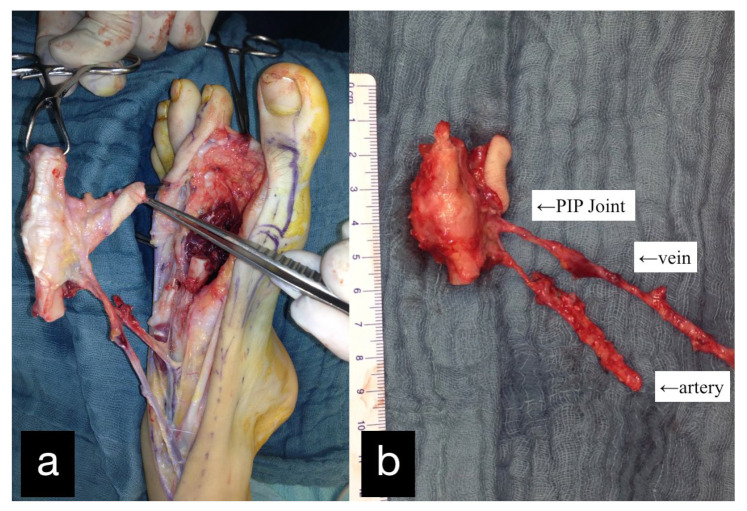
(**a**) Intraoperative PIP-Joint of the left second toe. (**b**) Explanted PIP-Joint of the left second toe with vein, artery, and skin island.

Three months after the first treatment, the MCP joint of the left index finger was reconstructed by transplantation of the PIP joint of the left second toe (Figure 2). 

Anastomoses, plate osteosynthesis, and the reconstruction of the extensor tendon were performed. 

**Figure 3:**
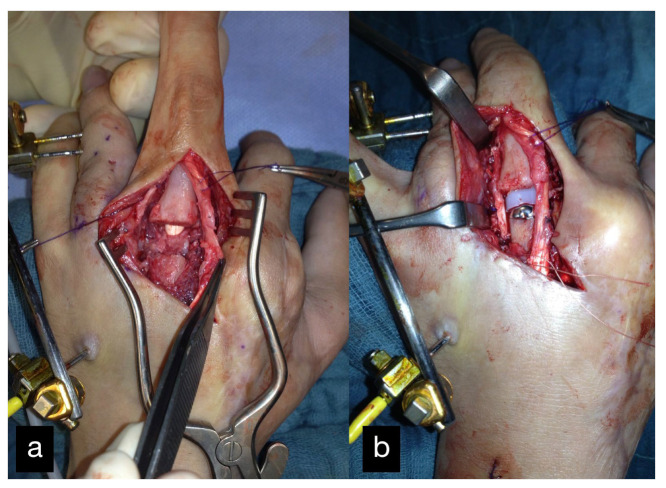
MCP joint of the middle finger before (**a**) and after (**b**) reconstruction using an MCP endoprosthesis.

Six months after the initial treatment, a DIII MCP endoprosthesis was installed (Figure 3).

**Figure 4:**
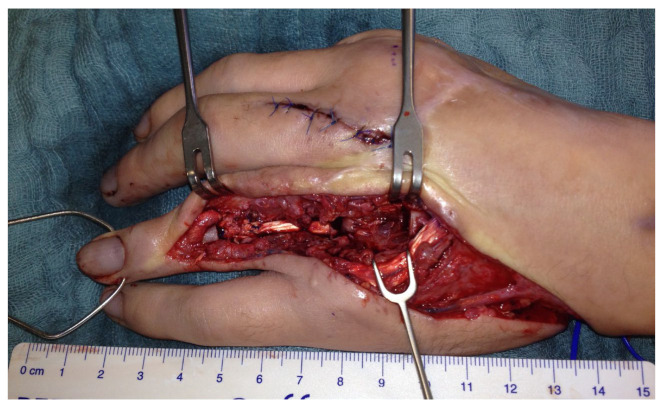
Left hand with demolished DIV PIP and MCP joints.

Another three months later, a free vascularized transfer of two coherent joints was carried out. The defect included the PIP and the MCP joint of the finger (Figure 4). 

**Figure 5:**
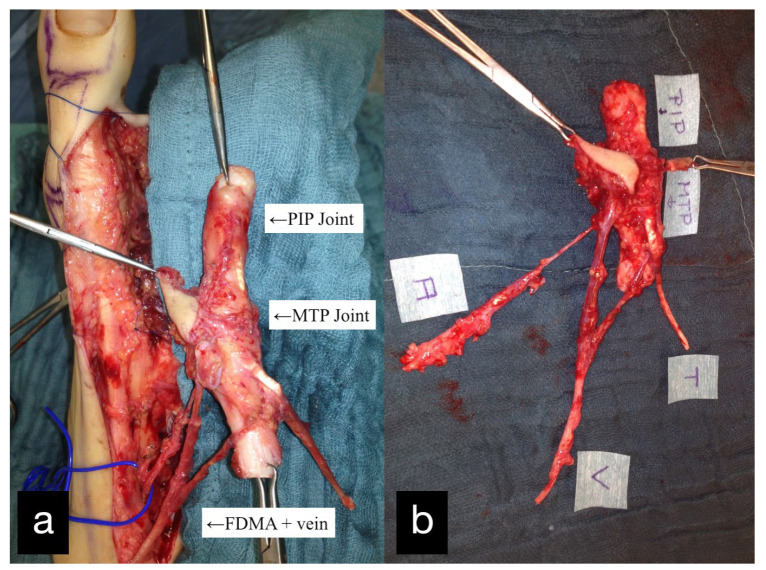
(**a**) Intraoperative PIP and MTP joints of the right second toe and first dorsal metatarsal artery and vein (FDMA + vein). (**b**) Explanted PIP and MTP joints with the tendon (T), artery (A), vein (V), and skin island.

The double joint from the right second toe, consisting of the MTP and PIP joint, was removed. The graft was pedicled on the first dorsal metatarsal artery including the vena comitans. A skin island was used for monitoring (Figure 5).

The reconstruction of the extensor tendon and the osteosynthesis were performed.

**Figure 6:**
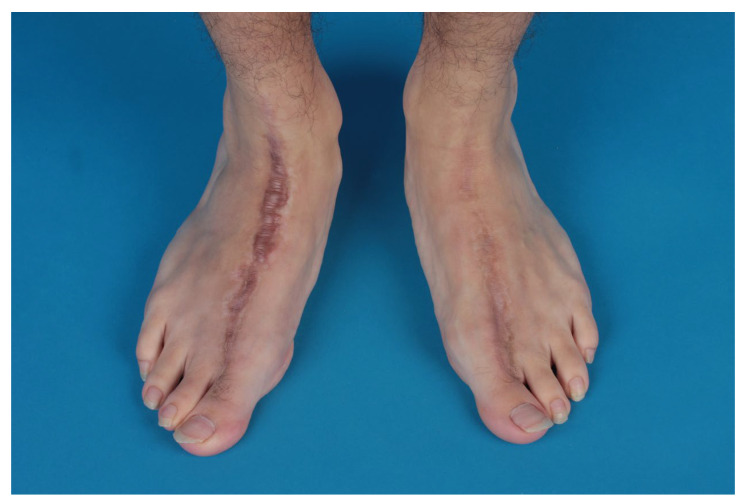
Both feet after harvesting and narrowing.

During the same operation, a narrowing of the foot was performed (Figure 6). 

**Figure 7:**
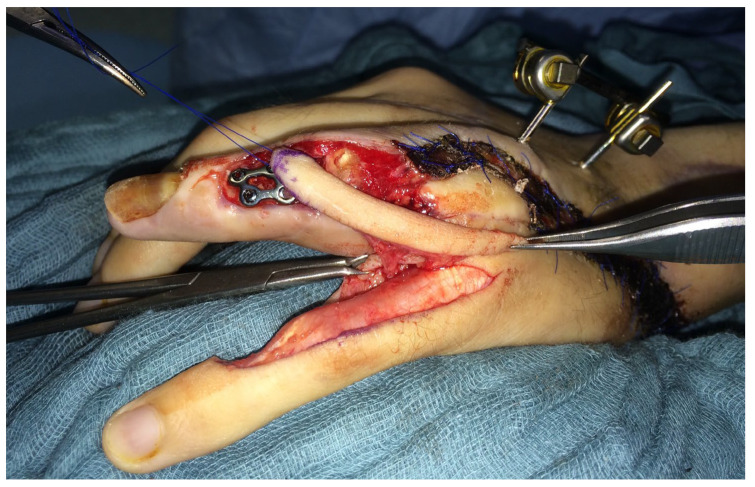
Heterodigital island flap pedicled on artery A9 to cover a defect over the exposed osteosynthesis of the ring finger.

Two weeks after toe transfer, a dorsal soft tissue defect of the ring finger occurred, and the osteosynthesis was exposed. A heterodigital island flap pedicled on artery A9 was performed to cover the defect (Figure 7).

Approximately 9 months after the joint transfer, the patient was subjectively symptom-free. The patient worked as an office administrator and the activities of daily life were possible without any problems.

Eleven months after the joint transplantation an X-ray showed minor deviation of the distal phalanx of DIV and a subluxation in the PIP joint replacement. 

We used an iliac crest bone graft to improve the minor malrotation and maintain length at the same time. 

One year after the joint transfer, a revision of the osteosynthesis with reosteosynthesis on the middle phalanx of DIV as well as scar correction was performed.

The final inspection took place 2 years after the accident.

There was good vascularization of the joint transfer, which was visible on the monitor islands. 

**Figure 8:**
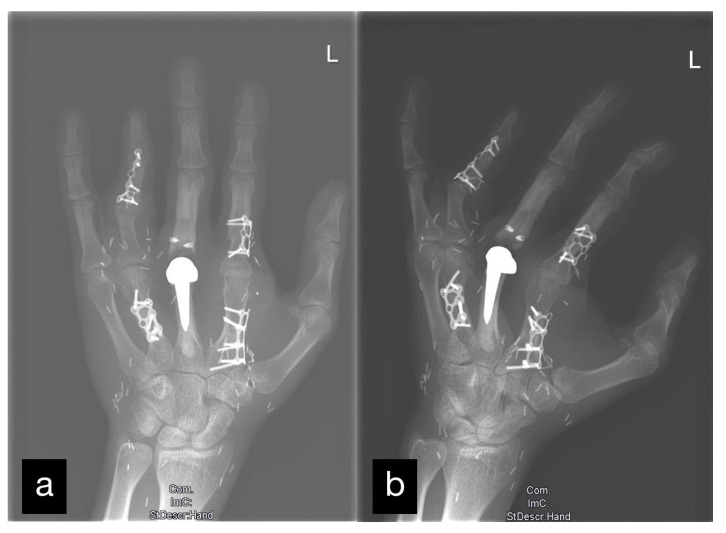
X-ray of the left hand after all mentioned reconstructions. Two levels: (**a**) a.p. and (**b**) oblique.

The joint space was preserved without changes typical for arthritis (Figure 8).

The active range of motion and the two-point discrimination were documented for each finger using the neutral zero method (Table 1). 

**Figure 9:**
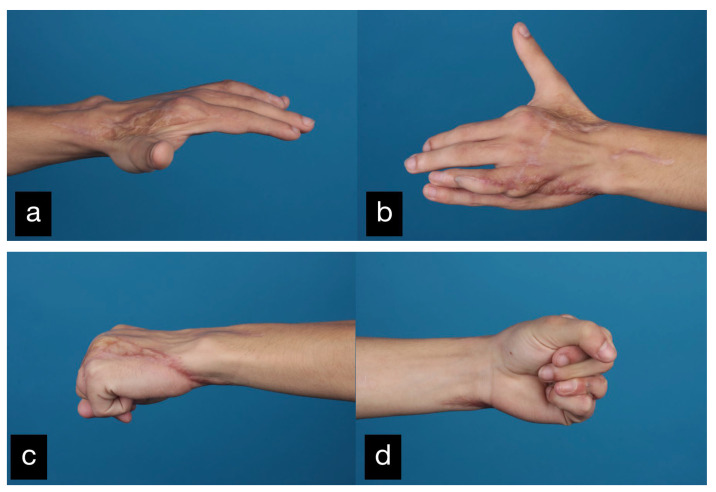
Final result displaying an active range of motion. (**a**,**b**) Extension of all fingers, (**c**,**d**) Fist closure.

The grip strength was approximately half of the contralateral right site. The final result displaying the active range of motion can be seen in Figure 9.

The radiological control showed that the osteosynthesis had been completely built up.

Both feet underwent narrowing and showed excellent cosmesis (Figure 6).

### 2.2. Patient 2

**Figure 10:**
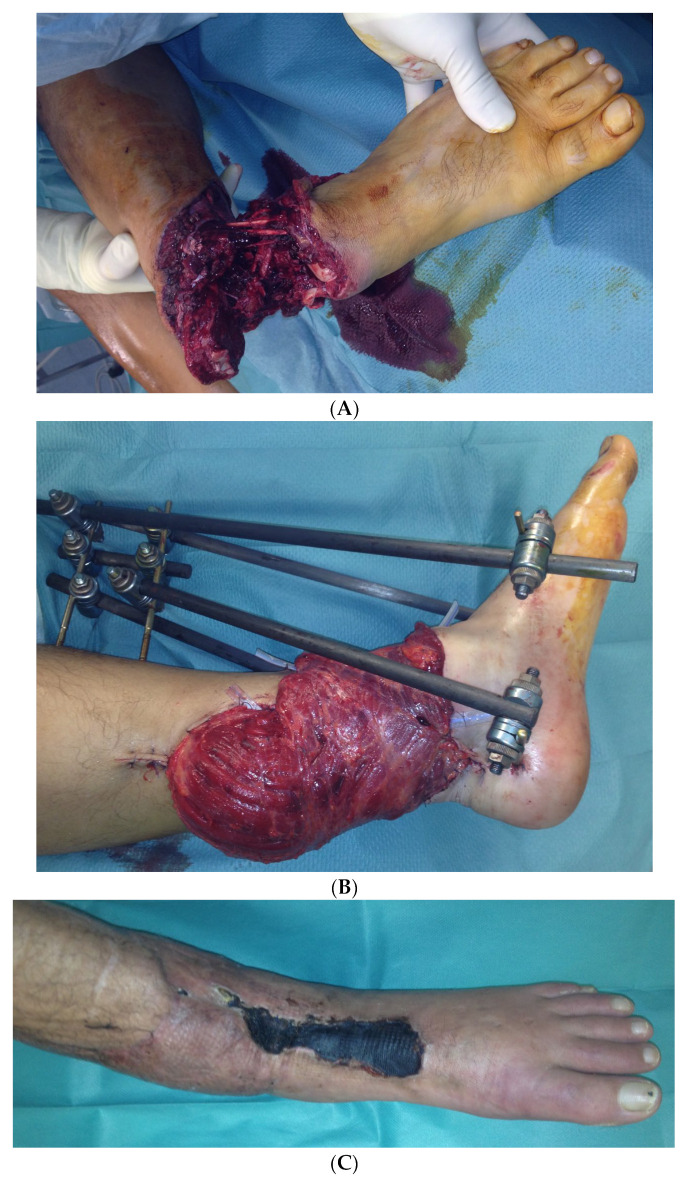

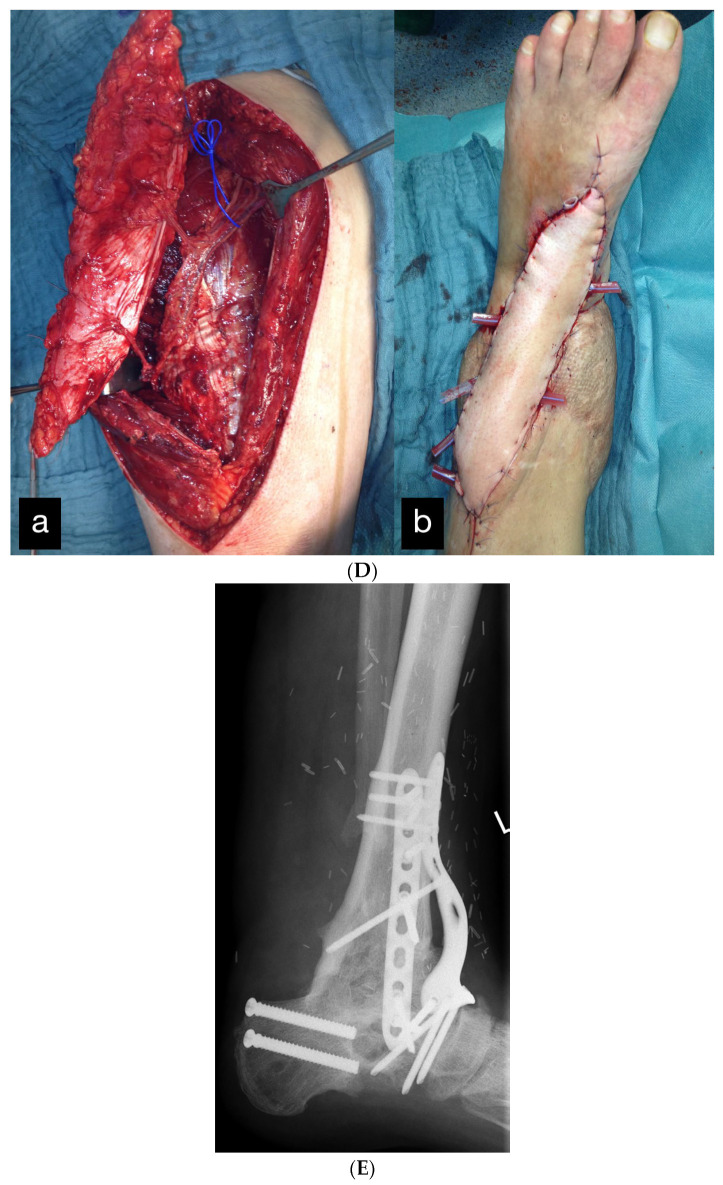

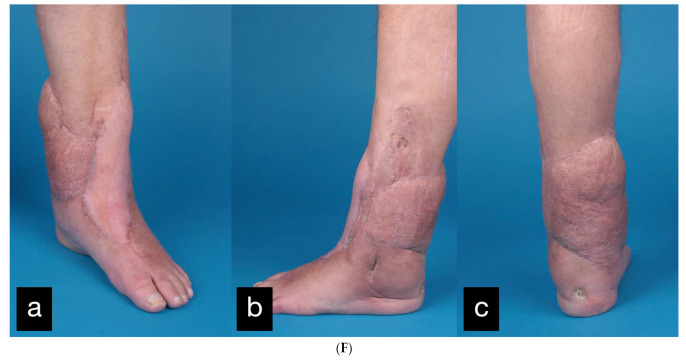
(**A**) Partial amputation of the left leg. (**B**) Postoperative result after a free latissimus dorsi flap to cover the soft tissue defect and an external fixator. (**C**) The 10 × 2 cm skin necrosis on the dorsum of the foot. (**D**) (**a**) Intraoperative free ALT flap of the contralateral leg. (**b**) Intraoperative transfered free ALT flap. (**E**) Double-plate compression arthrodesis with free vascularized iliac crest flap. (**F**) (**a**–**c**) Final inspection five years after the accident with completely built-up arthrodesis and a stable stand. Non-irritated scars and a free flap with good perfusion..

In the second case, a 45-year-old male patient was hit on the left lower leg by a steel beam. The partial amputation was only held by the tendons of the M. tibialis anterior and the Achilles tendon on the lower leg (Figure 10A). The subtotal lower leg amputation showed no peripheral blood flow, sensitivity, or motor function.

During the initial treatment, the anterior tibial artery was sutured using a vein interposition graft, four veins were supplied with an interposing suture, an anterior and posterior tibial tendon suture, and an external fixator attached to the left ankle, as well as a temporary soft tissue covering with alloplastic material. Five days after the accident, the soft tissues were covered with a free latissimus dorsi flap (Figure 10B).

Three months after the initial restoration, the fixator was removed and a nail arthrodesis of the left ankle was performed.

After four months, the patient could be discharged for outpatient follow-up care.

In the further course, a 10 × 2 cm skin necrosis occurred on the dorsum of the foot (Figure 10C) due to a compression of the tibial anterior artery by the arthrodesis screw. Both lesions were covered using an anterolateral thigh (ALT) flap (Figure 10D).

Because of the development of a pseudarthrosis of the left ankle, a double-plate compression arthrodesis with a free vascularized iliac crest was performed 3 years after the initial treatment. 

This was the third free flap on this foot.

The final inspection took place 5 years after the accident (Figure 10F).

The patient was completely symptom-free and reported a clear improvement in sensitivity in the sole of the foot, as well as deep sensitivity in the area of the flap. Radiologically, the arthrodesis was completely built up.

### 2.3. Patient 3

In the third case, an 18-year-old male patient was admitted after injuring his right hand while working with a metalworking machine.

**Figure 11:**
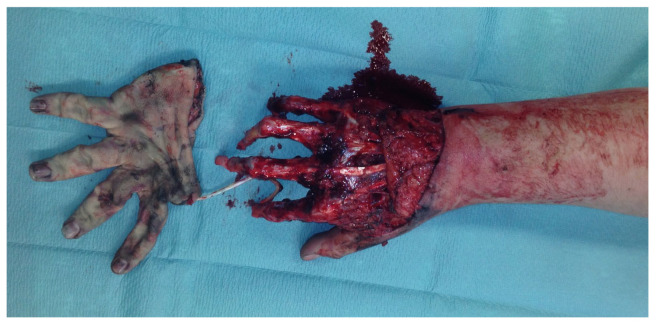
Avulsion of all long fingers of the right hand.

During the clinical examination, the soft tissue of all long fingers of the right hand was avulsed on the extensor and flexor side (Figure 11). 

In addition, there was a borderline amputation of DII and a disarticulation of the distal phalanx of DIII–DV. 

**Figure 12:**
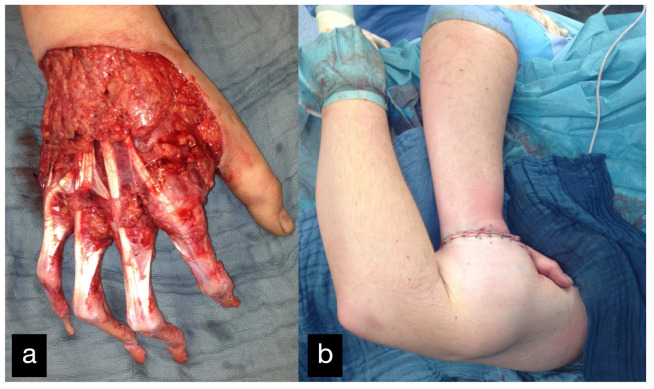

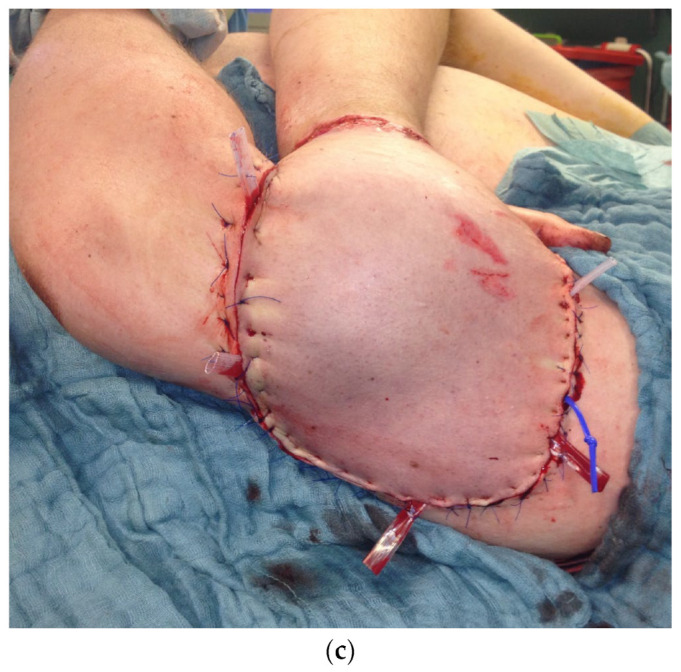
(**a**) Intraoperative picture of the right hand after debridement. (**b**) Collson flap with the right hand placed in the left upper arm. (**c**) Partial flap detachment after three weeks.

Because the peeled-off soft skin tissue was not suitable for replantation, a Colson-plastic was performed. The right hand was placed in the left upper arm (Figure 12a,b). Due to an existing obesity, the patient decided against a groin flap and in favor of positioning the hand on the upper arm.

In a further step 4 weeks after the Colson flap, the flap detachment was performed and a complete dorsal covering with tissue ensued.

In the course of infected wound conditions, serial debridements needed to be carried out.

With appropriate wound conditions, the defect was covered with a Serratus fascia flap. In addition, further borderline amputations were performed on DII–V with resection of the FDP tendons, flap thinning, and plastic displacement flaps.

Two months after the accident the syndactyly in the area of the index and middle fingers were separated.

**Figure 13:**
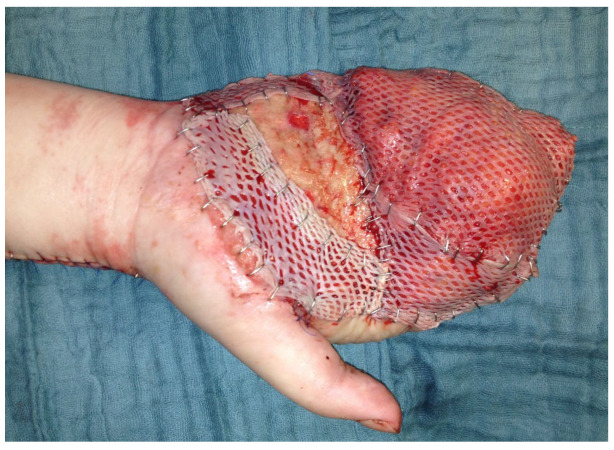
Serratus fascia flap with split-thickness skin grafts on the palm of the hand.

Because of poor perfusion of the middle and end phalanx, DII–DV had to be amputated in the process and the defect wounds were covered with split-thickness skin grafts from the thigh and with expansion flaps (Figure 13).

The patient was discharged 4 months after his accident. 

**Figure 14:**
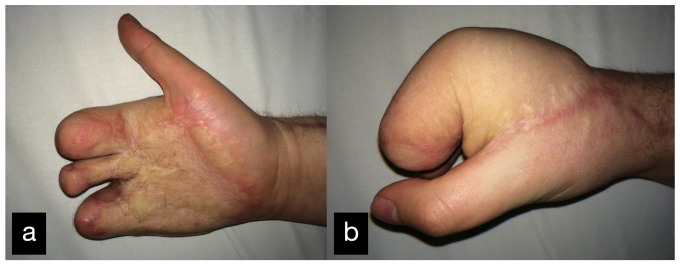
Final result displaying an active range of motion. (**a**) Full extension of the stumps, (**b**) Closure of the hand into a fist.

In a follow-up 3 months after the initial treatment, the patient stated that he was gradually coping with daily life and that he was able to open a lock with a key and eat independently with a knife and fork. The clinical examination showed very good mobility of the MCP joints with a deficit in extension of 25° and a possible flexion of 80 ° (Figure 14).

### 2.4. Patient 4

After a serious motorcycle accident, a 17-year-old patient was admitted as a polytrauma. In addition to multiple other injuries such as pelvic and thoracic trauma, abdominal trauma, and left femoral fracture with complex internal knee trauma, the patient suffered a complex injury to the left hand.

**Figure 15:**
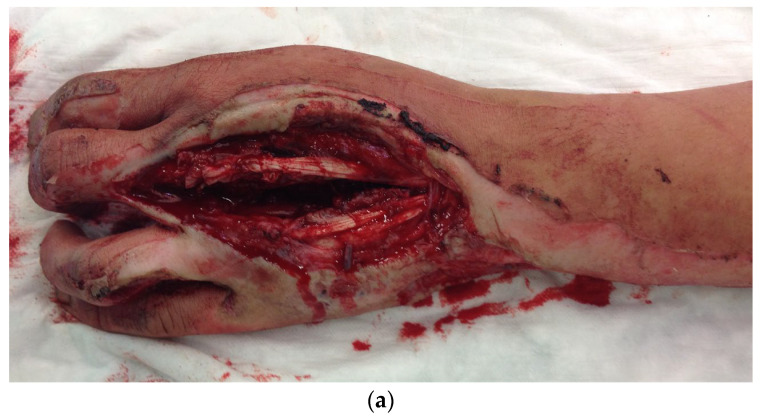

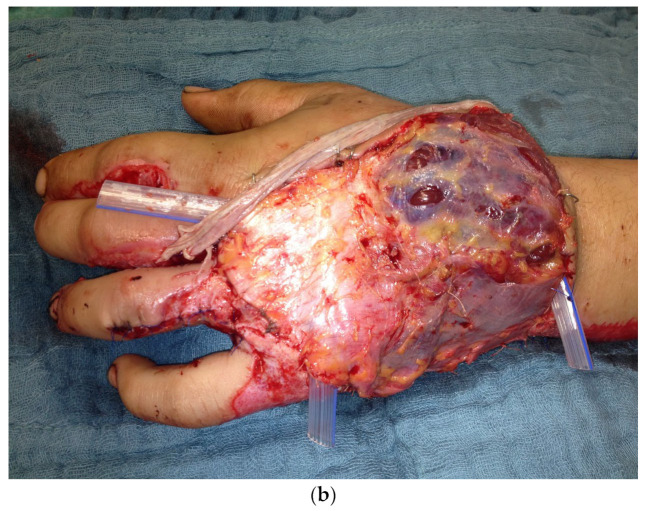
(**a**) Soft tissue defect on the back of the hand after a motorcycle accident. (**b**) Free fascia lata flap of the right thigh as soft tissue coverage and interposition graft.

There was a soft tissue defect on the back of the hand, a rupture of the metacarpal ligament and the extensor tendon of the ring finger (Figure 15a), 2a degree burns on the back of the hand, and 2b degree burns on the forearm.

Initially, the lesions of the ring finger were reconstructed with an interposition graft using the fascia lata of the right thigh. In addition, a soft tissue coverage using a fascia flap of the tensor fascia lata was performed (Figure 15b).

**Figure 16:**
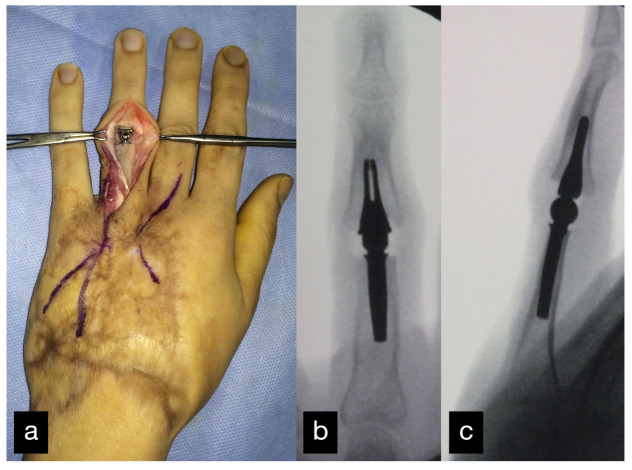
Replacement of the PIP joint of the left ring finger with a joint prosthesis. Intraoperative picture (**a**) and postoperative X-ray a.p. (**b**) and sideways (**c**).

Ten months after the initial operation, the PIP joint of the left ring finger and the MCP joint of the left middle finger were replaced with a joint prosthesis (Figure 16).

Due to a developed buttonhole deformity of the ring finger on the left, a correction was necessary 2 years after the accident with a reconstruction of the tractus intermedius using parts of the retinaculum extensorum.

Because of an infection of the prosthesis after several operations on the left hand, the PIP joint endoprosthesis on the left ring finger had to be explanted. The patient then presented selectively for arthrodesis using an iliac crest chip, after regression of the infection.

**Figure 17:**
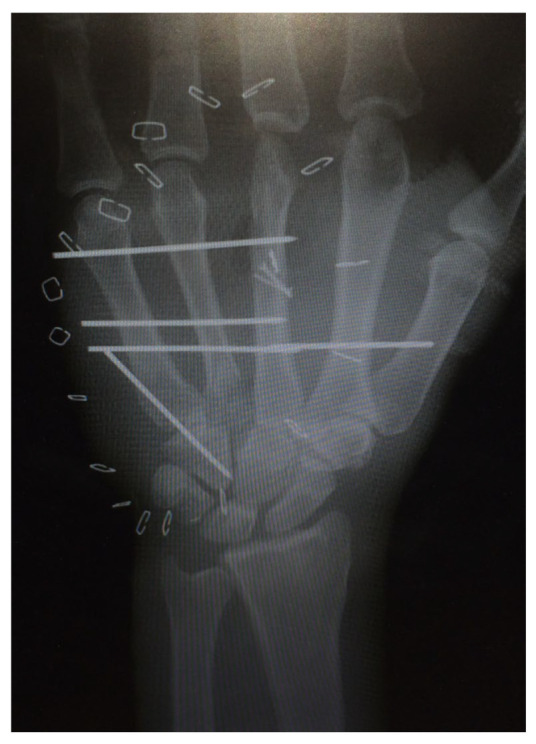
Radiological control after 1 year with correct position of the iliac crest graft and inserted arthrodesis material.

The radiological control after 1 year showed a correct position of the iliac crest graft and the inserted arthrodesis material (Figure 17).

**Figure 18:**
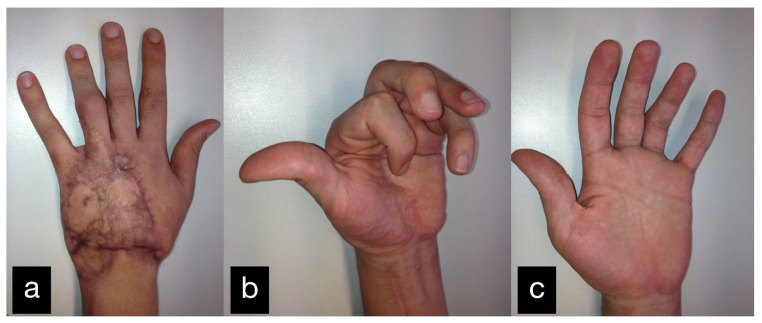
Final result displaying an active range of motion. (**a**) Extension of all fingers in dorsal view, (**b**) Maximum flexion of all fingers, (**c**) Extension of all fingers in palmar view.

In a follow-up the patient stated that he was gradually coping with daily life. The clinical examination showed a satisfying mobility (Figure 18).

### 2.5. Patient 5

In the fifth case, a 58-year-old male patient suffered a right lower leg fracture. Initial treatment was provided in an external hospital. The postoperative course of primary care was fraught with complications. Because of a broken implant and loosened osteosynthesis screws revision surgery was performed.

Reosteosynthesis using a plate and corticospongious iliac crest span was performed 6 months after initial care. The patient was treated with a double antibiotic therapy (levofloxacin and rifampicin) due to Staphylococcus haemolyticus colonization.

**Figure 19:**
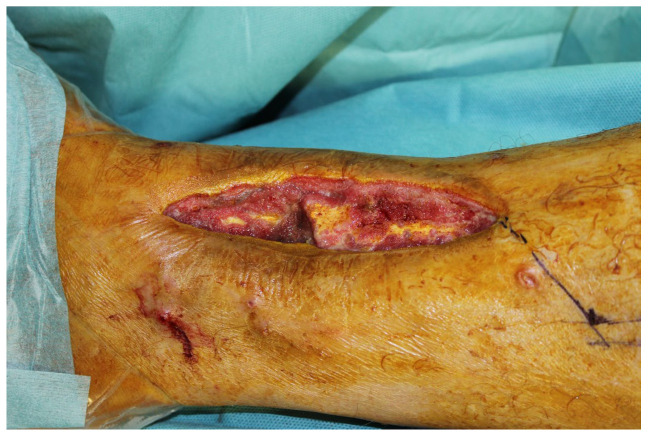
Infected pseudarthrosis of the distal tibia with implant dislocation and fistula formation.

We were first consulted 9 months after the accident when the patient was presented with an infected pseudarthrosis of the distal tibia with implant dislocation and fistula formation (Figure 19).

The clinical examination showed a fistula opening about 5 cm in diameter in the scar area with secretion. The indication for revision with removal of the material and a fistula debridement was made.

The CT performed at the first appointment showed a lack of osseous build-up of the lower leg fracture on the right with dislocation of the osteosynthesis material.

The osteosynthesis plates and screws were removed and a Lizarov-fixator was applied to the right distal tibia, as well as a VAC application.

**Figure 20:**
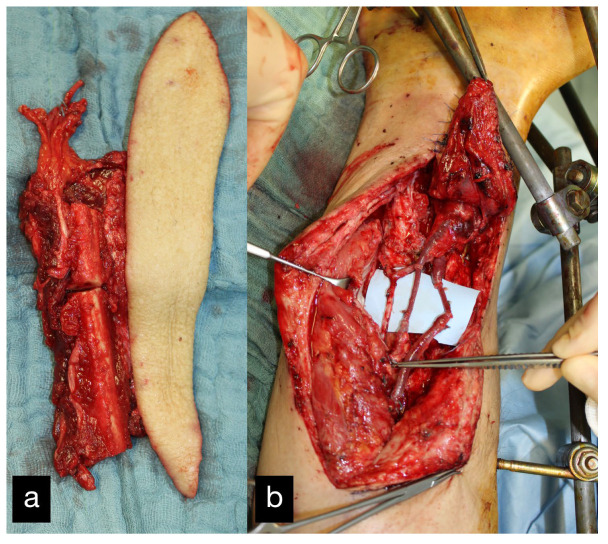
(**a**) Intraoperative osteofasciocutaneous free fibula. (**b**) Free fibula bolted into the defect zone. Vascular connection end to end to the posterior tibial artery proximal to the defect zone.

After several vacuum changes with extensive debridements, an osteofasciocutaneous free fibula transfer was performed from the left as a double barrel, which was bolted into the defect zone (Figure 20a). The vascular connection was made end to end to the posterior tibial artery proximal to the defect zone (Figure 20b). 

**Figure 21:**
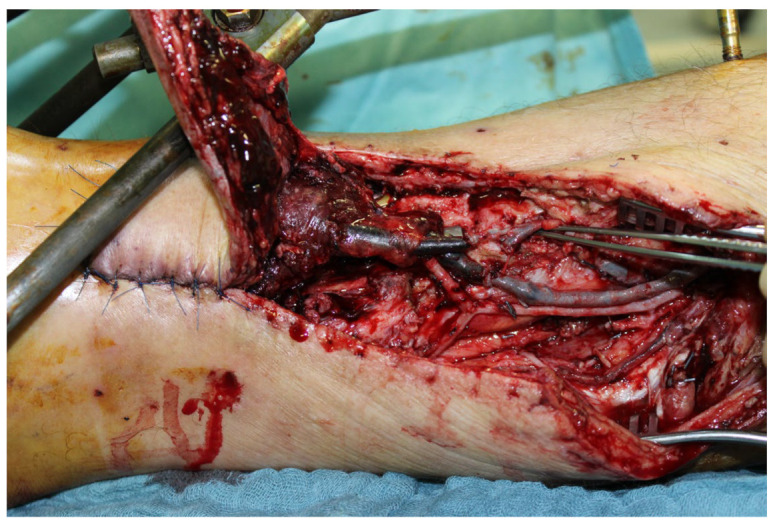
Thrombosis of the envois system on the first postoperative day.

Because of a livid discoloration on the first postoperative day a revision of the flap with reconstruction of the venous system using a vein interposition was made (Figure 21). A few hours later, the flap on the right lower leg was again lividly discolored; therefore, another flap revision was performed on the same day. Intraoperatively, there was another venous problem, and another vein interposition using the great saphenous vein was performed. Postoperatively, the right lower extremity was placed in a free-floating position and, due to the venous problem, was treated with leeches. In the further course, sonography showed again a thrombus in the venous system. Since the operative options had already been exhausted, it was decided to take a conservative approach using leech therapy and consistent elevation. In addition, due to a misalignment, there was a venous stasis with a subsequent livid discoloration. In the further course, superficially necrosis was removed and a split-thickness skin graft was done. Three months after the first consultation, the patient could be discharged for further ambulant treatment.

### 2.6. Patient 6

A 50-year-old male patient had an accident at work with a punching machine and suffered a subtotal amputation of the right hand with a fracture of the radius and ulna and a severing of all functional structures.

**Figure 22:**
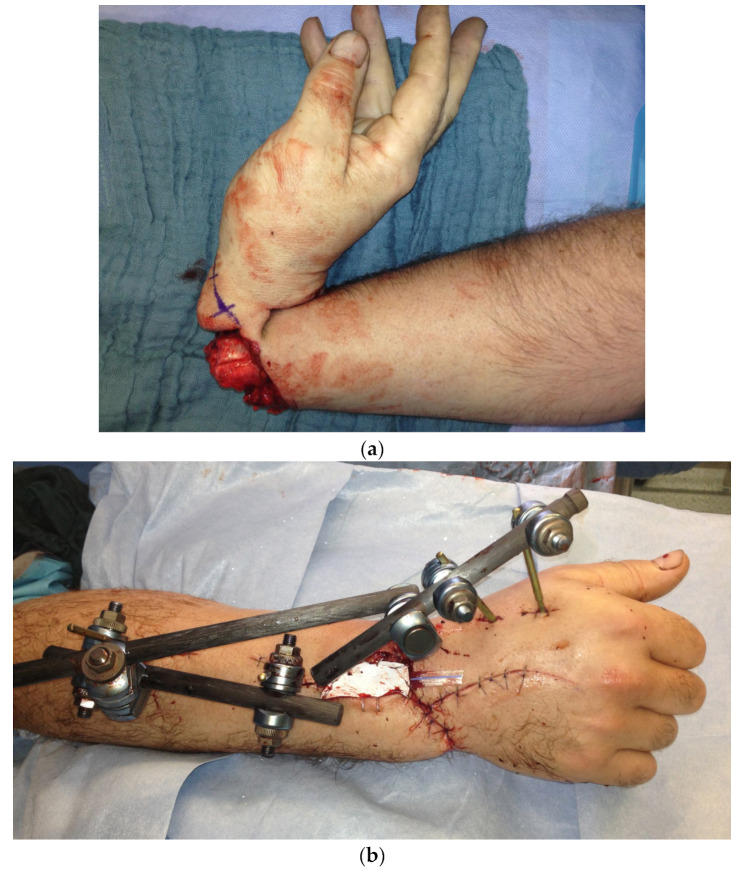

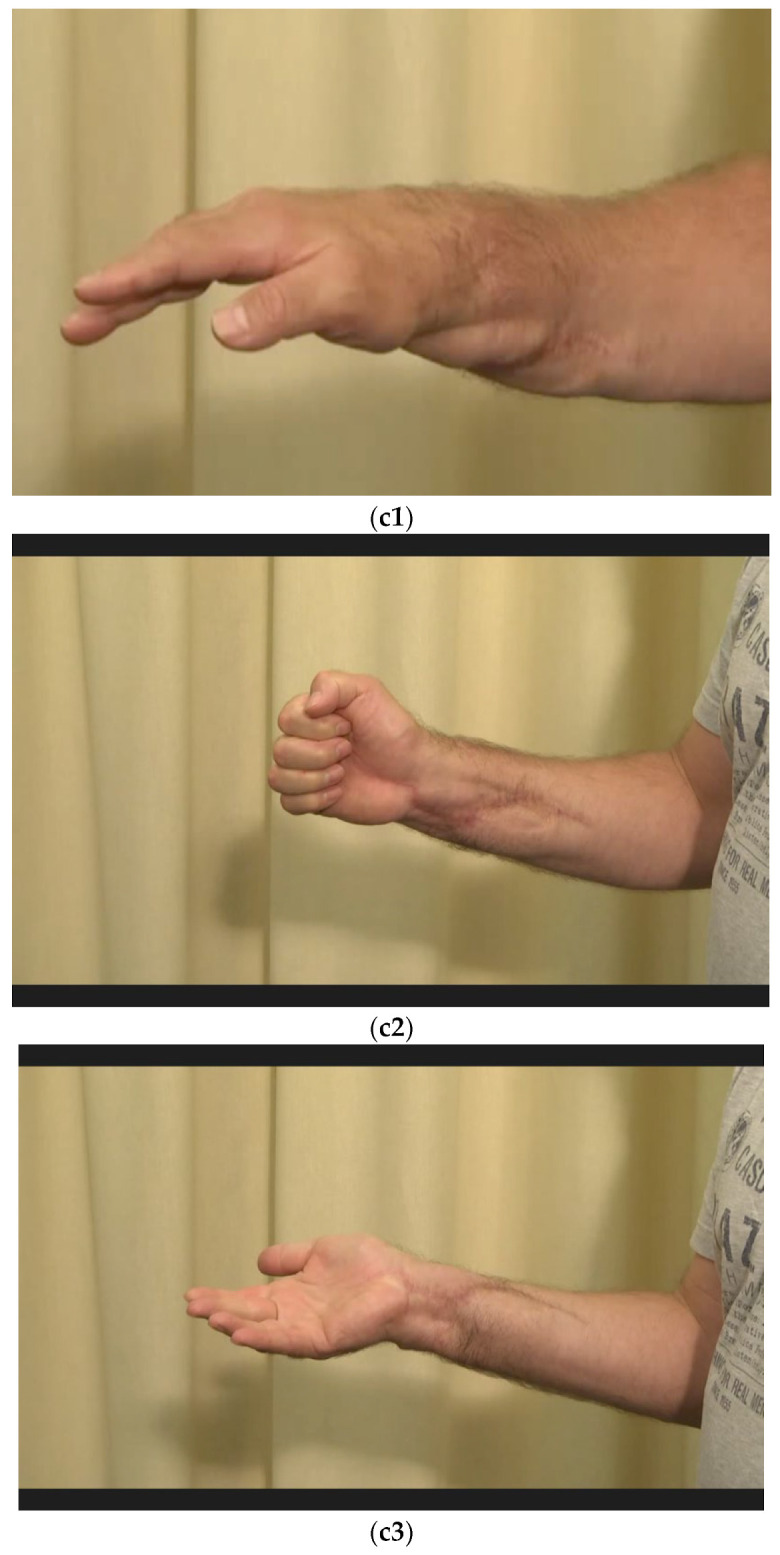
(**a**) Subtotally amputated hand at the level of the wrist with a third-degree open fracture of the radius. (**b**) Postoperative picture after anastomoses of the arteries, coaptation of the nerves, and reconstruction of all flexors and shown external fixator. (**c1**–**c3**) Final result displaying an active range of motion.

The clinical examination showed that the hand was subtotally amputated at the level of the wrist with a third-degree open fracture of the radius (Figure 22a). The radial and ulnar arteries were also severed. Due to a preserved skin bridge on the ulnar side, the peripheral circulation of the fingers was present.

Immediate surgical treatment with anastomoses of the ulnar and radial arteries, coaptation of the median, ulnar, and radial nerves, and reconstruction of all flexor and extensor tendons was performed. In addition, the radial joint surface was fixed with a Herbert screw, and the instability in the wrist was treated with an external fixator (Figure 22b). 

The soft tissue was covered both by an initial split skin graft from the right thigh to the right forearm, as well as by a temporary covering of the free deep-lying structures with alloplastic material.

Residual dorsal and volar defects could be treated with split skin after increasing granulation. The complete severing of all tendons on the extensor and flexor sides was immediately provided with an inverted Kleinert splint for dynamic exercise treatment.

The patient could be discharged after three weeks. 

Three years after the initial treatment, the patient presented restricted mobility in the area of the right thumb. The Kapanji score was 4/10. 

The clinical examination showed a two-point discrimination of 6 mm in all fingers. 

Intraoperatively, the APL tendon and the EPB tendon were strongly fused, which was corrected through tenolysis.

After physiotherapeutic follow-up treatment, thumb opposition was improved. The contact of the thumb end phalanx and the ring finger end phalanx was possible. The little finger could not be reached actively. Passively, there were no restrictions of motion (Figure 22(c1–c3)).

### 2.7. Patient 7

**Figure 23:**
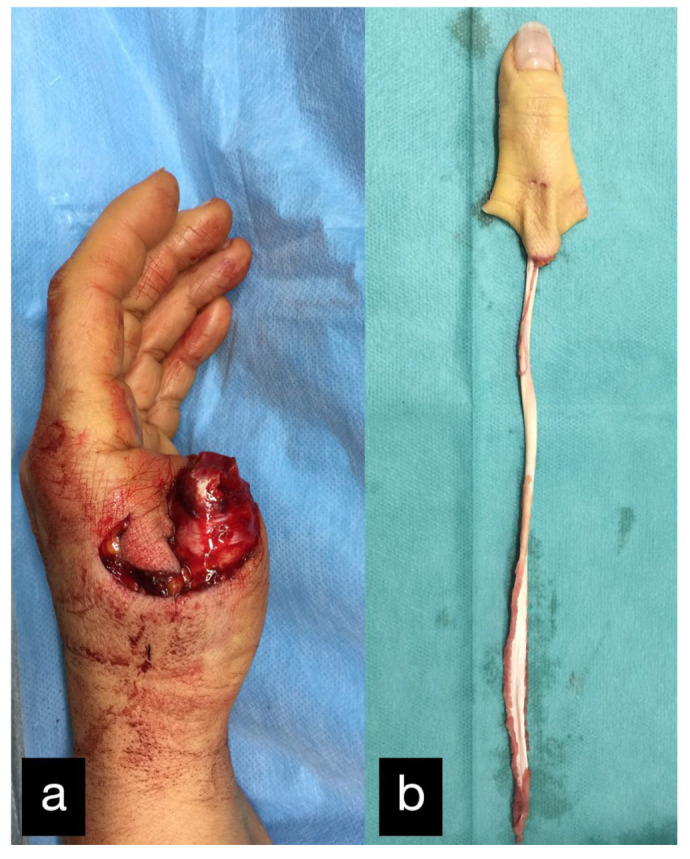
(**a**) Thumb amputation at the level of the basal phalanx of the left hand. (**b**) Amputated thumb with extensor tendon.

A 53-year-old patient was admitted after a traffic accident with a pelvic fracture and a thumb amputation at the level of the basal phalanx of the left hand (Figure 23).

**Figure 24:**
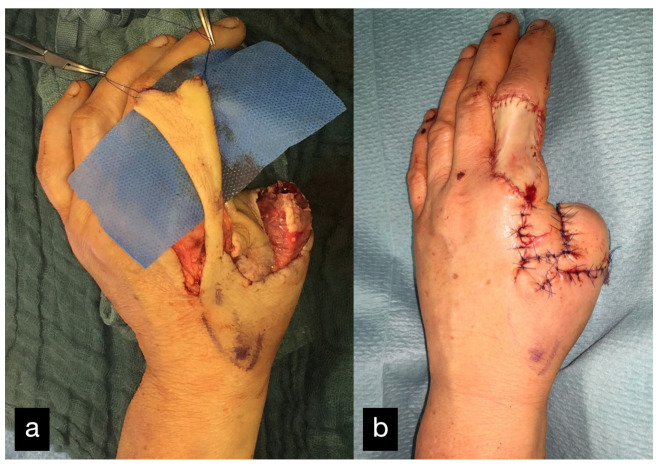
(**a**) Soft tissue covering over the remaining metatarsal stump, using a heterodigital flap of the dorsal index finger phalanx. (**b**) Postoperative picture with good perfusion of the flap.

Because of the increased blood loss due to the pelvic fracture, replantation was initially not possible. In the course of the process, to preserve the length of the amputated thumb, a soft tissue covering over the remaining metatarsal stump, using a heterodigital flap, was performed (Figure 24). A replantation was refused by the patient at this point.

The donor site was the area of the dorsal index finger phalanx in the sense of a DMCA flap. The donor site was covered with full skin.

The flexor pollicis longus tendon was torn up and could not be reattached in the area of the thumb. A silastic rod was therefore inserted consecutively into the fibroosseous canal.

**Figure 25:**
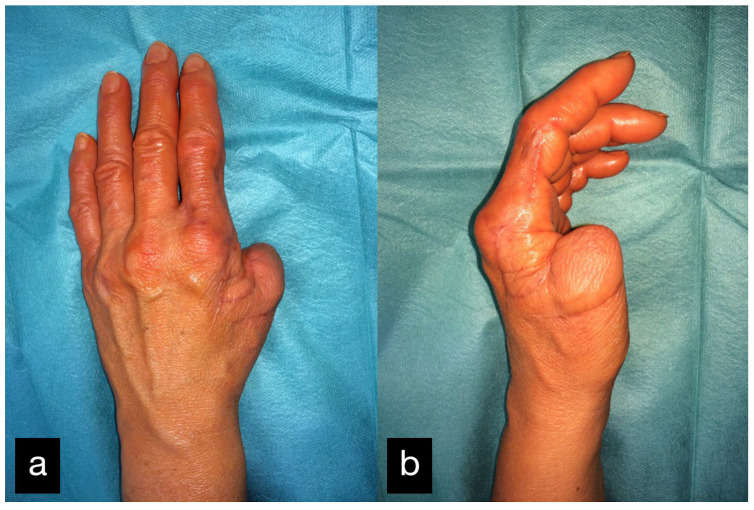
Final result displaying an active range of motion of the thumb (**a**) dorsal view, (**b**) radial view.

In the further course, the stump was non-irritated (Figure 25); however, the young active woman wanted a functional replacement.

**Figure 26:**
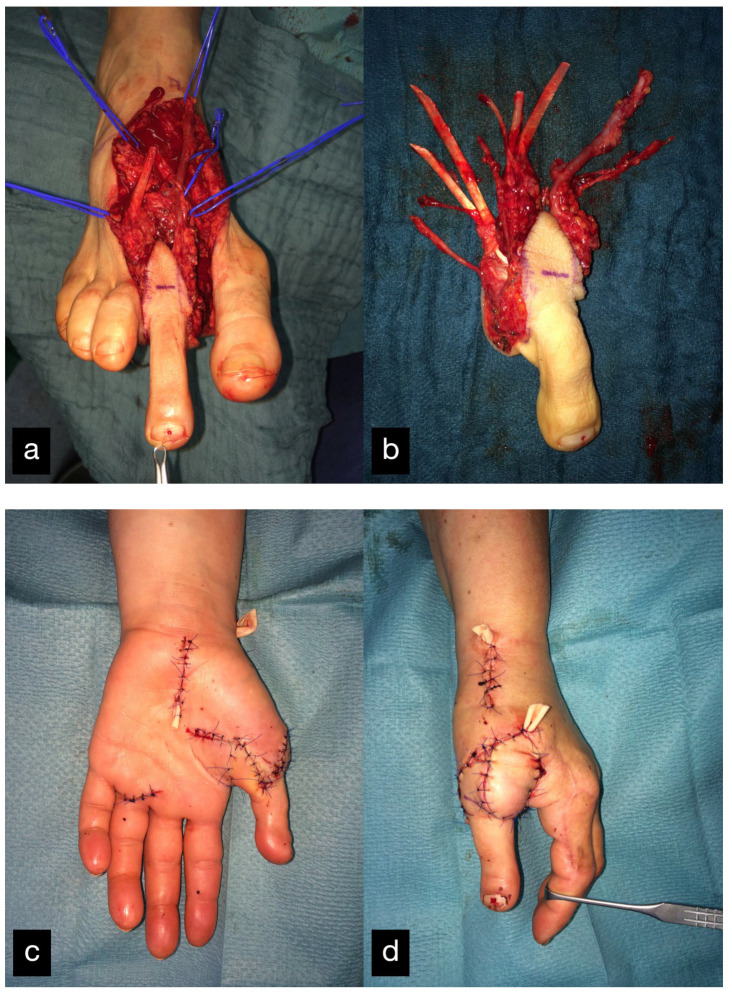
(**a**) Intraoperative harvested second toe of the right foot. (**b**) Explanted second toe with artery, venous system, and tendons. (**c**) Postoperative result palmar. (**d**) Postoperative result sideways.

**Figure 27:**
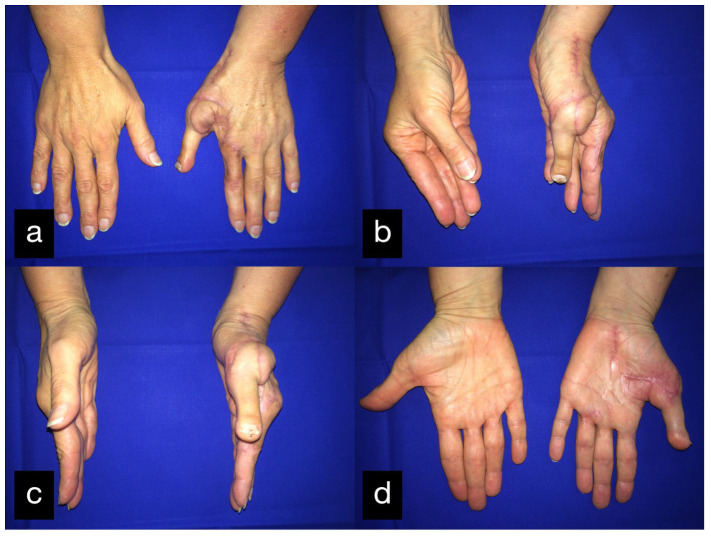
Final result displaying active range of motion. (**a**) Extension of all fingers in dorsal view, (**b**) Opposition of the thumb, (**c**) Abduction of the thumb, (**d**) Abduction of the thumb in palmar view.

Two months after the initial treatment, the second toe was transplanted as a thumb replacement (Figure 26). The final result displaying the active range of motion can be seen in Figure 27.

### 2.8. Patient 8

An 18-year-old male patient presented with an amputation of the left hand and a complex multi-level injury of the left forearm due to an auger conveyor.

**Figure 28:**
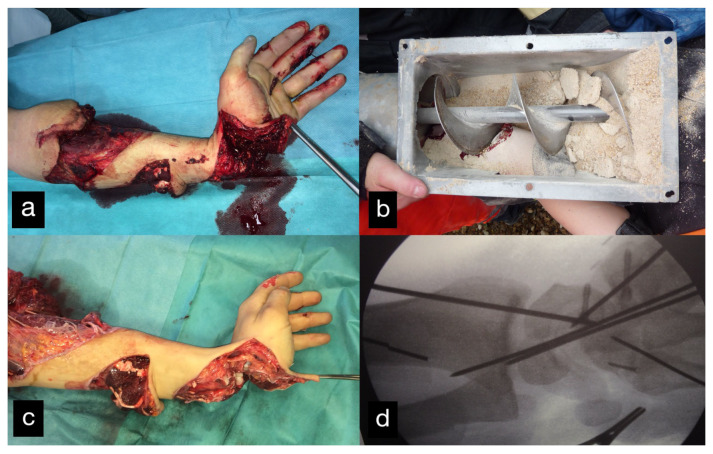
(**a**) Multi-level amputation of the left hand. (**b**) Auger conveyor. (**c**) Situs after debridement. (**d**) Osteosynthesis of the scaphoid and the trapezoid using Herbert screws, screw osteosynthesis of the os hamatum. Transfixation of the carpus.

The left hand was amputated at the level of the midcarpal with isolated preservation of the extensor tendons and a preserved skin bridge. The hand was avascular and also showed III° burns. There was a midcarpal dislocation and a fracture of the trapezoid, capitate, hamate, and of the distal scaphoid pole. The N. medianus was transected at the level of the wrist, the N. ulnaris at the level of the wrist, and at the level of the distal forearm. A. radialis and A. ulnaris were divided at the level of the wrist. The flexor tendons were injured at several levels (Figure 28a–d).

**Figure 29:**
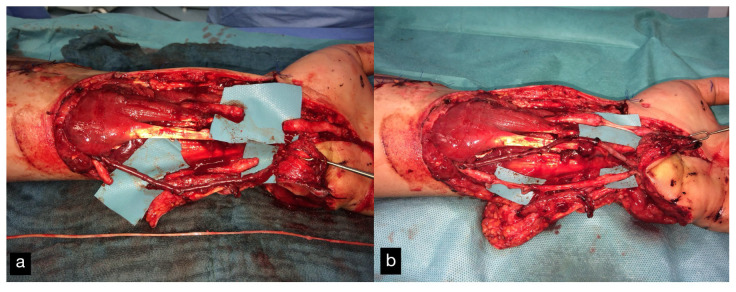
(**a**) Defect of the N.medianus and N. ulnaris. At the bottom of the picture, the n suralis can be seen as an interposition graft. (**b**) A. ulnaris with a vein interposition graft, N. medianus, and N. ulnaris with N. suralis interposition grafts.

A replantation with reconstruction of all injured structures at every level was performed. In addition, an osteosynthesis of the scaphoid and the trapezoid was performed using a Herbert screw and a screw osteosynthesis of the os hamatum. Transfixation of the carpus using five wires (Figure 28d). The A. ulnaris required a vein interposition graft. N. medianus and N. ulnaris were reconstructed using N. suralis as interposition grafts (Figure 29). The soft tissue was covered with a temporary soft tissue replacement. Three days postoperation, the defect was covered with a free ALT-flap and a split-thickness skin graft.

After insufficient flexor tendon preservation of the thumb, the tendons of the flexor pollicis longus, the flexor pollicis brevis, and the extensor pollicis longus were reconstructed again.

Three months after the accident, there was a persistent flexion deficit in all fingers, which is why tenolysis with additional neurolysis and a preparation of the arteries was performed.

The patient then received special hand therapy rehabilitation.

**Figure 30:**
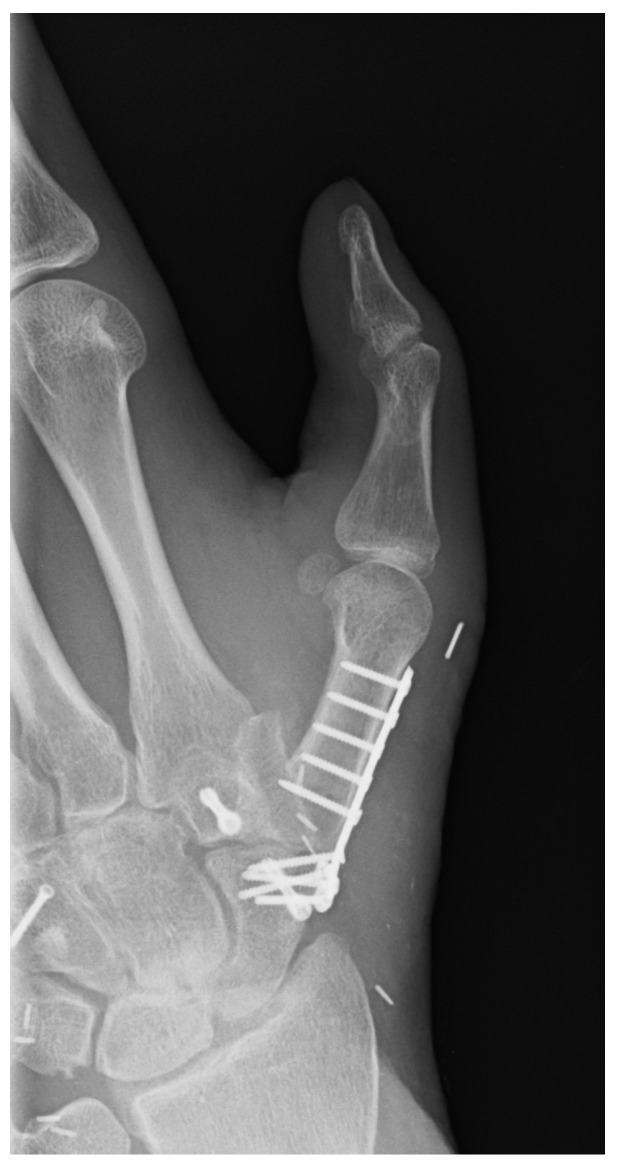
Complex three-dimensional corrective osteosynthesis and arthrodesis of the left thumb saddle joint.

Due to a persistent misalignment of the thumb, a complex three-dimensional corrective osteosynthesis and arthrodesis of the left thumb saddle joint were performed one year after the accident (Figure 30).

Sixteen months after the accident, the flap was thinned using liposuction and lipectomy.

Due to a fall, the patient sustained a subcapital fracture of the fifth metacarpal and a fourth metacarpal fracture near the base 3.5 years after the accident, which was treated with intramedullary K-wire splinting.

**Figure 31:**
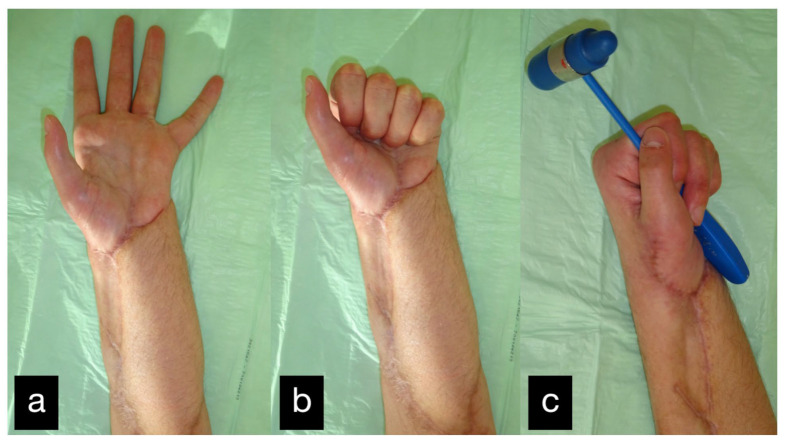
Final result displaying active range of motion. (**a**) extension of all fingers, (**b**) making a fist, (**c**) holding a hammer with satisfaying force.

The range of motion at the follow-up can be seen in Table 1 (Figure 31a–c).

After the final treatment, the patient was able to return to his agricultural training.

## 3. Discussion

To achieve a good functional result, the initial reconstruction of all the important structures is necessary. 

It is well known that most patients have more than one secondary procedure [2,4,5,38,45].

The tissues encountered include skin, soft tissue, tendon, nerve, bone, and joint [7]. 

Surgical techniques for secondary procedures do not vary greatly from standard procedures but are technically more difficult [46,47].

The timing and order of the performed secondary surgery is important, and only compatible procedures should be performed simultaneously. Because of the postoperative immobilization that is required for soft tissue reconstruction, bone reconstruction, and nerve repair, procedures like tenolysis should not be performed simultaneously. In return, tenolyses, arthrolyses, or arthroplasties may be performed at the same time [2].

Most of the literature mentions secondary procedures in the reports of functional results after replantations [9,10,11,15]; however, few papers have focused on this topic [12,13,14].

**Timing of secondary reconstruction:**

The timing of secondary surgery is determined primarily by the clinical course after replantation. 

Therefore, continuous monitoring by experienced hand surgeons and therapists is needed. 

For the initial secondary procedure, it is best to wait until improvements of joint, nerve, and tendon function decrease significantly [2]. 

This plateau, during therapy, usually occurs about 4 to 6 months after the replantation [11]. 

Other conditions like bone nonunion, skin defects, or malunion should be treated immediately [48]. 

Some authors divide secondary procedures into early and late groups and chose an interval of 2 months after replantation, excluding the procedures for vascular complications. In this period, almost all the initial therapy to achieve survival of the replanted body part is completed. The secondary work will not be performed by 3 to 6 months following replantation [7].

The most common surgeries in the early groups were procedures for treatment of the unhealed open wound. In the late group of secondary procedures, tendon treatment was most common [7].

Interestingly, younger patients had more late secondary operations than elder patients [7].

**Order of secondary reconstruction:**

Using the largest review of replantation cases available, Wang et al. provide a stepwise approach for determining the order and the need of performing secondary procedures [43].

This algorithm uses an anatomical basis in that patients need soft tissue reconstruction first. Then, skeletal reconstruction and nerve reconstruction is needed, not only for stability but also to enable sensitivity in the finger. Afterwards, joints are reconstructed and only after that can the most frequently needed procedure, which is the reconstruction of tendons, be performed. 

Fortunately, most patients will not require all of the steps previously mentioned [43].

**Secondary soft tissue reconstruction:**

The fundamental reconstructive principle of restoring the soft tissue coverage is to perform surgery with a result that is both aesthetically acceptable and durable to enable early and sustained functional rehabilitation.

Since the reconstructive ladder is no longer performed the simpler surgical procedures are skipped, this is referred to as the reconstructive elevator [49] or also called reconstructive rocket [50].

More extensive defects with exposure of underlying structures often require pedicled or free flap in the form of fasciocutaneous, muscle-only myocutaneous flaps or are designed in chimeric fashion [51]. The use of flaps is preferred because only they allow the tendon gliding [43]. An often overlooked consideration is the problem of free flap re-elevation in situations where secondary reconstruction or revision is needed, fasciocutaneous flaps allow easier secondary flap elevation [51,52]. This point should always be incorporated into the reconstructive plan [51]. Another point that should always be in mind is the fact that a lot of free flaps, especially used on the hand, have to undergo multiple debulking procedures [53].

If allowing secondary wound healing or using split-thickness grafts sometimes causes joint or webspace contracture. Full-thickness skin grafts undergo less contraction. Soft tissue contracture of the first webspace is also common after thumb replantation because of simple scarring or contracture of the adductors.

If needed, a contracture release is performed within a secondary surgery procedure. Small contractures or first webspace contractures can be released with a Z-plasty. Large contractures or contractures with only one plane of supple skin need a Y-V advancement flap or have to be resurfaced by a transposition flap or a microvascular free flap from remote donor sites [2,54].

Another common contracture is a flexion contracture; when skin contracture coexists, the replantation scar should be incorporated into a Bruner-type incision [55]. 

**Secondary skeletal reconstruction:**

Initial fixation methods contribute to the incidence of postreplantation nonunion or malunion, crossing-over, or angular-rotational deformities of replanted digits [16,48].

Nonunion is recognized immediately after 4 to 8 weeks after replantation. Achieving bony union through surgery can reduce splinting time and thus facilitate therapy, resulting in a better functional outcome. 

There are different techniques for secondary skeletal reconstruction. 

If the resection of nonunion results in a shortening of the digit, then that is not acceptable for the patient, and a bone graft is indicated [2].

The deformity can be corrected when first noticed, or later when functional improvement makes deformities visible. To correct the angulation or reduce the rotation, a corrective osteotomy may be needed. 

Realignment is followed by bony fixation, and bone grafting is rarely indicated [2]. 

If a revision is not recommended because of the complexity of the original fracture or because of the soft tissue coverage, a corrective osteotomy at a more proximal level may be considered [48].

**Secondary nerve reconstruction:**

Initial direct treatment remains the current standard for the repair of peripheral nerve lacerations; however, 5.4% of all secondary operations are nerve-related procedures [56]. This includes neurolyses, neurectomy, neurorraphies, or interpositional nerve grafts because of inadequate initial treatment [56].

Poor function, symptomatic neuromas, or pain/hypersensitivity of a digital nerve should lead to a revision. These operations should be considered only after the stabilization of soft and skeletal tissue. 

The preparation of neurovascular bundles should start proximal to the level of the lesion because of unexpected positions after replantation [2].

For lesions with large nerve gaps, conventional sural nerve grafting remains the most viable option [57]. Another option is nerve scaffolds including autologous conduits, artificial nonbioabsorbable conduits, and bioabsorbable conduits [57,58]. Reconstruction with vein conduit grafts also gives excellent results [59] in secondary digital nerve reconstruction.

Some authors present a retrospective study to evaluate the role of the free fasciocutaneous flap and the importance of sensory nerve reconstruction in improving long-term results [60]. This shows the possibility of treatment with an innervated flap for large soft tissue lesions with large nerve gaps or missing nerves. 

**Secondary joint reconstruction:**

After soft tissue, skeletal, and nerve reconstruction, the next step is joint operations if needed. 

After the replantation of body parts, e.g., fingers, secondary procedures like capsulotomy, capsuloplasty, arthroplasty, or even the free vascularized transfer of joints are used.

Capsulotomies, because of joint contractures need careful analysis to isolate the dorsal and volar components of the problem [2].

If deformities of the finger such as swan necks, mallet, and intrinsic minus deformities [11]—because of the elongation of the injured tendon—appear after replantation, capsuloplasty is necessary. Capsulorrhaphy is indicated to correct significant deformities.

Fingers with arthrodesis during replantation need arthrodesis replacement, using silicone joint prostheses to produce functionality [18,61]. Swanson prostheses are the standard implants in these cases [62].

Although arthrolysis is often required, the need to reconstruct a joint completely is rare [63]. Despite prostheses, there is also the possibility of osteochondral grafts and microvascular joint transfers [64,65].

The free microvascular transplantation of a toe joint is indicated in children and active patients, especially when multiple joints are damaged. Performing free vascularized transfer of toe joints leads to a normal anatomic structure [66]. The combined arc of motion using a double transfer of toe joints shows a better range of motion in comparison to just one transferred joint [67].

In the case of a free vascularized transfer, the joint can be used straight or upside down. 

In the upside-down technique, the joint is turned 180° around its longitudinal axis, because the motion of the MCP joint is mainly hyperextension. 

Following the reversed implantation, it allows better mobility [68,69,70].

Although the choice of donor toe is debatable, the second toe is usually used. However, due to its similarity and stability, it is recommended to use the big toe to reconstruct the thumb [71,72].

**Secondary tendon reconstruction:**

Stiffness after complex injuries or a long time of immobilization is common because of the multiple injured structures that are involved. For some patients with problems of this kind, hand therapy may be useful, but others need tendon operations to improve the function [2]. 

Tendon procedures should be performed only when skin, skeletal, nerve, and joint functions are restored. 

Secondary operations include tenolysis with or without joint capsule release, tendon grafting, and pulley reconstruction. Moreover, 70.2% of delayed tendon procedures are tenolysis procedures to release tendon adhesions, whereby flexor tendon tenolysis is the most common late secondary procedure [7,73].

Extensor tendon tenolysis and capsulotomy are scheduled when the active and passive range of motion is limited.

If a tendon is ruptured, thinned out, or bridged with scar tissue, a staged tendon reconstruction should be performed with a silicone tendon rod and a final reconstruction with tendon grafting [74,75,76].

Amputation as secondary surgery after replantation is rarely reported [11]; however, reamputation can sometimes be a logical choice due to tendon adhesions, infection, necrosis or gangrene, nonunion or malunion, pain or hypersensitivity, scar tissue contractures, or joint destruction [56,75].

**Reamputation:**

Reamputation accounts for about 16.7% of secondary procedures [56,75].

Late amputations are performed just proximal to the most proximal stiff joint. For psychological and social benefit, the stump can be adapted to digital prostheses. Sometimes it is the patient’s wish to perform a reamputation because of a prolonged wound healing [2].

But there are also cases where a ray resection should be considered. This decision requires careful consideration and detailed consultation with the patient because a narrowed palm reduces twist grip strength [76]. This secondary procedure is necessary if the stump of the index finger hinders the pinch between the thumb and middle finger or if a gap in the transverse palmar arch is created due to an amputated middle or ring finger [2].

**Figure 32:**
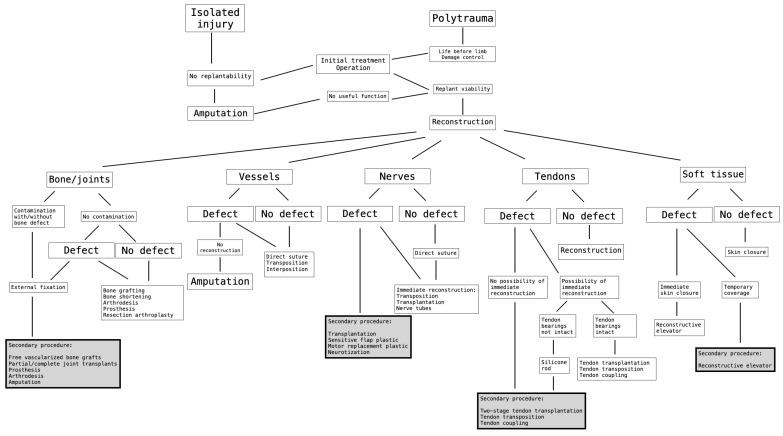
The decision-theoretic approach for determining the order of performing secondary procedures after complex limb trauma [2,43].

Based on Wang et al. [2] and the above-mentioned six major cases, we followed the algorithm, which is shown in (Figure 32). 

## 4. Conclusions

Treatment of complex trauma should be measured by functional outcome; therefore, secondary surgical procedures are necessary to improve the final result.

Patients should be informed of the possible need for subsequent surgery to improve the outcome.

We present a case series of patients with secondary complex microsurgical reconstructions after amputation and severe trauma injuries with a complicated post-operative treatment. 

We described the use of a decision-theoretic approach to the secondary reconstruction following the descriptions of Wang et al. [2].

Following this algorithm, we demonstrated an improvement in patient-reported disabilities. The patients gained a satisfying functional outcome.

To confirm this algorithm and its efficacy in practice, and to better understand the causal relationship behind secondary procedures, further research in the clinical setting is required.

## Figures and Tables

**Table 1:** Detailed information about the patients, clinical condition, reconstruction, and outcome.

Case Nr.	Age	Sex	Clinical Condition	Reconstruction in Chronological Order	Functional Outcome	2PD/Sensitivity	Grip
1	17	m	Circular saw injury to the dominant left hand with a subtotal amputation of DII–DIV at the level of the MCP joints	Replantation of DIV Amputation of the distal metacarpals DII and DIII, PIP-Joint transfer to DII from the left second toe, MCP endoprosthesis DIII, Free double toe joint transfer to PIP and DIP from the right second toe	Finger-palm distance (FPD) DII–DV 0–0–1–0 cm Fingernail table distance (FNTD) DII–DV 0–0–0–0 cm Kapanji 10/10. DII: MCP 0–0–80°, PIP 0–0–100°, DIP 0–0–80°; DIII: MCP 0–0–80°, PIP 0–0–100°, DIP 0–0–50°; DIV: MCP 0–10–90°, PIP 0–0–90°, DIP 0–0–10°; DV: MCP 0–0–90°, PIP 0–0–100°, DIP 0–0–90°.	The two-point discrimination reached 4–6 mm in all fingers.	Pinch left 7 kg, right 10 kg. Force left 8 kg, right 18 kg.
2	45	m	Amputation of the left lower leg because of trauma caused by a steel beam	Initial replantation, Free latissimus dorsi flap, Free ALT flap for ventral defect arthrodesis OSG with vascularized iliac crest	Full weight bearing possible. No ROM OSG because of arthrodesis.	Improvement in sensitivity in the sole of the foot, as well as deep sensitivity in the area of the flap	-
3	18	m	Avulsion of the right hand due to a metalworking machine. Amputation of DII and disarticulation of the distal phalanx of DIII–DV	Colson plastie left upper arm, Free serratus fascia flap for palmar hand, Amputation of DII–V	Mobility of the MCP joints: 0–25–80°	No data	No data
4	17	m	Motorcycle accident with pelvic and thoracic trauma, abdominal trauma, left femoral fracture with complex internal knee trauma. Soft tissue defect on the back of the hand, rupture of the metacarpal ligament and extensor tendon of DIV, 2a degree burns on the back of the hand, and 2B degree burns on the forearm.	Free fascia lata interposition graft, Joint prosthesis PIP DIV and MCP DIII, Arthrodesis PIP DIV using an iliac crest chip	Kapanji 9/10 FNTD DII–DV 1–1–2–0 cm FPD DII–DV 1.5–5–6–4 cm	No data	No data
5	58	m	Right lower leg fracture, infected pseudarthrosis of the distal tibia with implant dislocation and fistula formation	Osteofasciocutaneous free fibula transfer	No data	No data	-
6	50	m	Subtotal amputation of the right hand at the level of the wrist with a third-degree open fracture of the radius	Anastomoses of the ulnar and radial arteries, coaptation of the median, ulnar, and radial nerves, and reconstruction of all flexor and extensor tendons	FNTD DII-DV 1.5–2–2–1.5 cm FPD DII–DV 0-0-0-0 cm Kapanji 4/10	The two-point discrimination reached 6 mm in all fingers	No data
7	53	w	Traffic accident with a pelvic fracture and a thumb amputation at the level of the basal phalanx of the left hand	Heterodigital flap, second toe transplantation	FNTD und FPD DII bis DV je 0 cm, Kapanji 10/10	No data	No data
8	18	m	Amputation of the left hand and complex multi-level injury of the left forearm	A. ulnaris reconstruction with vein interposition graft. N. medianus and N. ulnaris reconstruction with N. suralis interposition graft. Free ALT flap	FPD DII-DV 0–0–0–0 cm FNTD DII-DV 2–2–2–2 cm. Kapanji 5/10. Range of motion of the wrist: Extension/flexion 30–0–40°. Supination/Pronation 90–0–90°	Two-point discrimination: DI–III 6 mm, DIV 6–8 mm and DV 8 mm	No data

## Data Availability

The original contributions presented in the study are included in the article, further inquiries can be directed to the corresponding author.
